# Supplementary material for: Viral non-coding RNAs hijack host Pumilio proteins to regulate host transcripts
Source: bioRxiv. 2025 Dec 1:2025.11.27.691032. Preprint. [Version 1] doi: 10.1101/2025.11.27.691032 (PMC12687793; doi:10.1101/2025.11.27.691032)
Supplement: 1 [file NIHPP2025.11.27.691032v1-supplement-1.pdf]

bioRxiv preprint doi: https://doi.org/10.1101/2025.11.27.691032; this version posted December 1, 2025. The copyright holder has placed this preprint (which was not certified by peer review) in the Public Domain. It is no longer restricted by copyright. Anyone can legally share, reuse, remix, or adapt this material for any purpose without crediting the original authors.

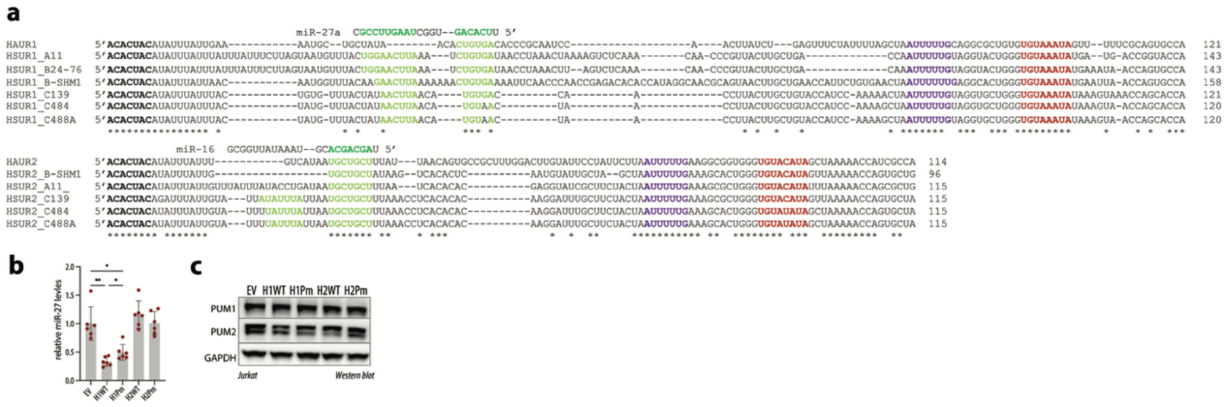

Figure S1. Related to Fig. 1. (A) PREs are conserved in HSUR1 and HSUR2 across multiple HVS isolates and related herpesvirus ateles. **(B)** Quantification of miR-27a levels normalized to miR-20; *p ≤ 0.05 and **p ≤ 0.01 by paired t-test. **(C)** PUM1 and PUM2 levels are not affected by the presence of HSUR variants. Western blot analysis of protein levels in Jurkat cells transduced with the indicated variants.

bioRxiv preprint doi: https://doi.org/10.1101/2025.11.27.691032; this version posted December 1, 2025. The copyright holder has placed this preprint (which was not certified by peer review) in the Public Domain. It is no longer restricted by copyright. Anyone can legally share, reuse, remix, or adapt this material for any purpose without crediting the original authors.

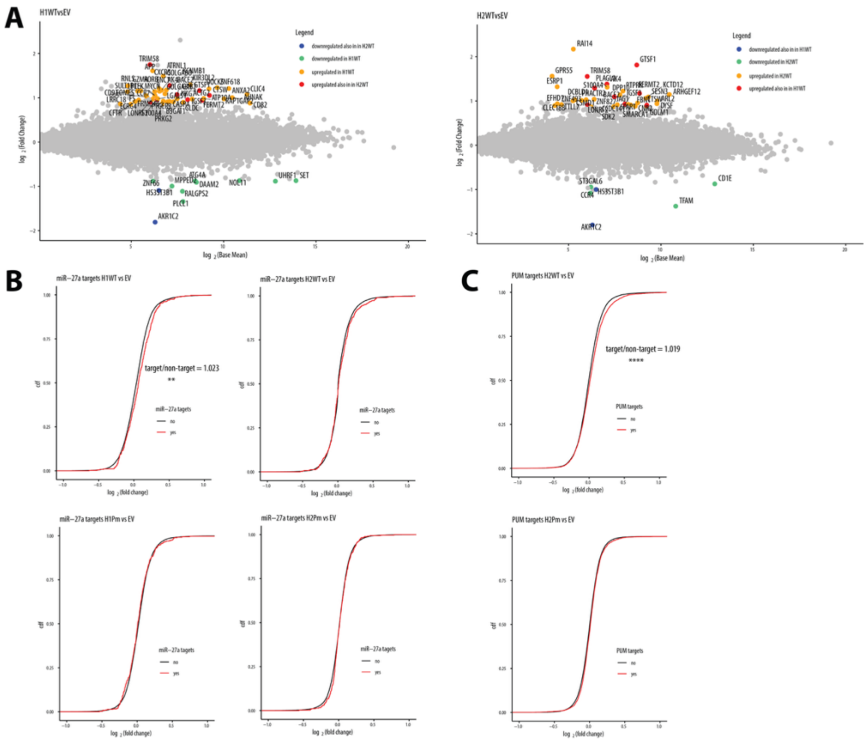

Figure S2. Related to Fig. 2. (A) Impact of WT HSURs on RNA levels in Jurkat cells. Shown are transcripts changing at least 2-fold with an adjusted p-value ≤ 0.05 (n = 3). **(B)** miR-27a targets exhibit a global increase in the presence of HSUR1, but not the other HSURs, as shown by cumulative distribution plots of log_2FC for miR-27a targets (from TarBase[55]) versus non-targets (**, p-value from Wilcoxon test). **(C)** HSUR2 WT leads to a global stabilization of PUM targets, as shown by cumulative distribution plots of log_2FC for PUM targets versus non-targets (****, p-value from Wilcoxon test). FC fold change.

bioRxiv preprint doi: https://doi.org/10.1101/2025.11.27.691032; this version posted December 1, 2025. The copyright holder has placed this preprint (which was not certified by peer review) in the Public Domain. It is no longer restricted by copyright. Anyone can legally share, reuse, remix, or adapt this material for any purpose without crediting the original authors.

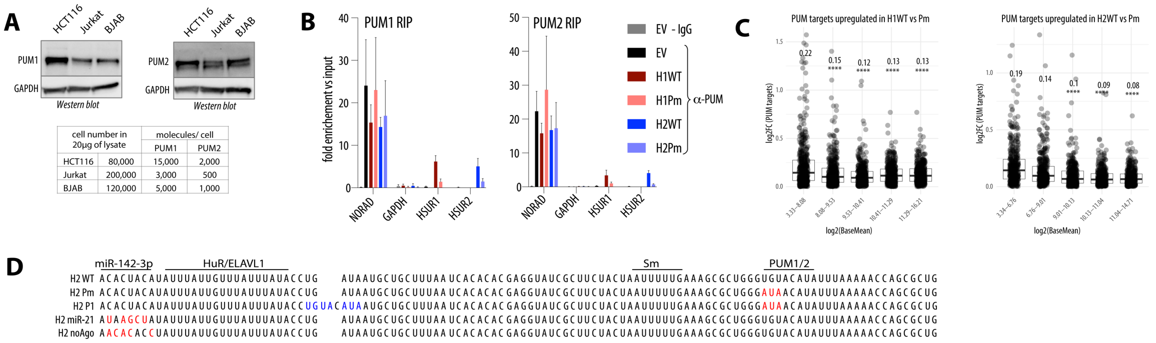

Figure S3. Related to Fig. 3. (A) Estimated numbers of PUM1 and PUM2 protein molecules per Jurkat cell relative to published values for HCT116 cells. **(B)** RNA immunoprecipitation in Jurkat cells using anti-PUM1 (left), anti-PUM2 (right), or IgG control, shown as enrichment over input; mean of 3 independent experiments ± SD. **(C)** Less abundant PUM targets are more strongly regulated by HSURs in a PRE-dependent manner; PUM targets were binned by expression level and PRE-dependent changes in expression are shown for each bin. **(D)** Schematic of HSUR2 mutants designed to identify additional elements involved in regulation of PUM-dependent targets.

bioRxiv preprint doi: https://doi.org/10.1101/2025.11.27.691032; this version posted December 1, 2025. The copyright holder has placed this preprint (which was not certified by peer review) in the Public Domain. It is no longer restricted by copyright. Anyone can legally share, reuse, remix, or adapt this material for any purpose without crediting the original authors.

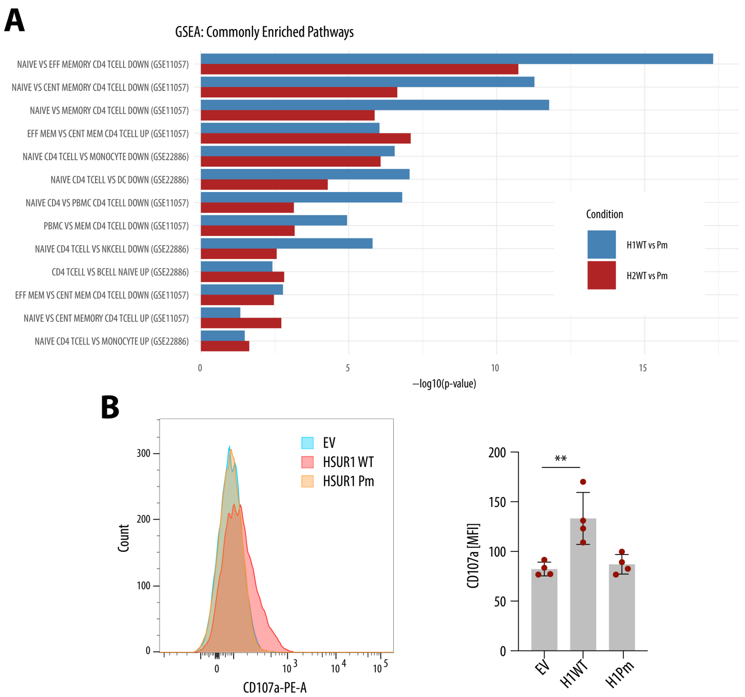

Figure S4. Related to Fig. 4. (A) Figure S4. Related to Fig. 4. (A) GSEA was restricted to MSigDB immunologic signatures containing "TCELL" in the gene set name and, for $CD4^{+}$-focused analyses, further filtered to sets containing "CD4"; shown are gene sets altered by both WT HSUR1 and HSUR2 compared with their respective Pm mutants. **(B)** WT HSUR1 increases CD107a surface levels upon PMA stimulation; a representative plot and quantification of CD107a MFI from three independent experiments are shown (mean ± SD; $p \le 0.01$, paired t-test). MFI, mean fluorescence intensity.

bioRxiv preprint doi: https://doi.org/10.1101/2025.11.27.691032; this version posted December 1, 2025. The copyright holder has placed this preprint (which was not certified by peer review) in the Public Domain. It is no longer restricted by copyright. Anyone can legally share, reuse, remix, or adapt this material for any purpose without crediting the original authors.

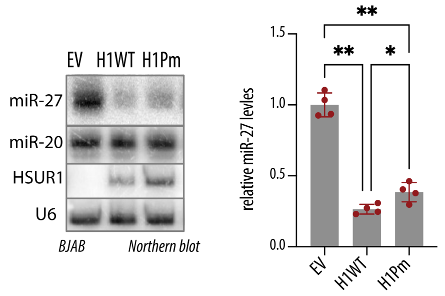

Figure S5. Related to Fig. 5. PRE mutation does not affect HSUR1 levels but slightly decreases TDMD of miR-27a. Representative Northern blot of RNAs from BJAB cells transduced with the indicated HSUR1 variants or empty vector (EV), probed for ncRNAs.
